# Common-Sense Dietetics

**Published:** 1911-12-16

**Authors:** 


### COMMON-SENSE DIETETICS.

By a most unfortunate chain of circumstances the authors of books upon dietetics have hitherto been divisible into two classes: the ultra-scientific chemico-physiological, with whom analyses *in vitro* and in calorimeters have been the paramount considerations; and the totally unscientific faddists, each with his own special obsession of some dietetic freak which shall cure all bodily ills. For the man who can steer clear of indiscriminating adherence to the former, while duly appreciating the meaning of many of the important facts brought to light by those who have investigated the physiology and psychology of digestion—and can estimate at their true worth the diverse follies and inconsistencies of the latter—for such a man there should be a large audience of ordinary people, willing and anxious to listen to some common-sense advice and to lay it to heart.* Mr. Leipoldt has gone to the best authorities for his physiology, and he has been amply receptive of the modern researches which show how largely digestion is influenced in the human stomach by factors quite outside the scope of test-tube experiments; that is by appetite, fatigue, age, condiments, and environment in general. He has also scant sympathy with the extremists, whose extravagances he has little difficulty in proving to be scientifically untenable.

So far then, the author has qualifications which may be called negative; that is, rather, he lacks the disqualifications of so many who have preceded him as writers on this topic. But besides this he combines a number of more positive virtues, which give his book a real distinction. Thus his acquaintance with the literature, ancient and modern, native and foreign, is extensive, even encyclopædic. His first-hand knowledge of foreign cookery seems to include that of nearly every quarter of the globe. His style is clear and lucid, his arrangement orderly and systematic. Common-sense is indeed the keynote of the book: and it is refreshing to meet an author who combines this valuable quality with scientific accuracy without doing violence to either. But there is more yet to place to Mr. Leipoldt's credit. Not only is he a born writer, but he would seem to be a born diner also. Anyone who reads through his chapters must see that his enthusiasm is that of the true *gourmet*; or, rather, in his own language, of the gastrosoph. Though we dislike the harshness of this word, which is neither euphonious nor etymologically pleasing, its definition by the author is worth quoting: "The gastrosoph is neither a *gourmand* nor a *gourmet*. He is a person who, having carefully studied dietetics and the art of eating, pays the greatest attention to the general principles, while at the same time allowing himself the widest latitude in their application to his habits of dining. He is the dietetic artist, the expert who knows how to eat, when to eat, and what to eat; who never permits convention to interfere with his appreciation of a tasty and wholesome dish, and despises the dictates of fashion that attempt to make him sit down to a meal which he cannot relish. He can appreciate vegetarian cookery when it is good, just as much as he can mixed cookery; he can abstain from caviare when it is dry and black without a pang, and dine off a Spanish onion and a bit of Parmesan cheese and black bread when he feels the inclination to do so. His dietetic life is regulated by moderation, sanity, discrimination, and controlled taste, not by arbitrary maxims laid down by authorities." The author has sampled the wines and the cookery of practically every corner of Europe, and of many more distant parts. His chapter on the art of dining shows that he has profited by his travels, and is worthy of study, not only by those who aim at being gastrosophs, but by everyone who wishes to round off his knowledge of common-sense dietetics.

* *Common-Sense Dietetics.* By C. Louis Leipoldt, F.R.C.S. (London: Williams and Norgate. 1911. Pp. 243. Price 2s. 6d. net).

